# Diagnosis and Management of Cervical Thymic Cysts in Children

**DOI:** 10.7759/cureus.973

**Published:** 2017-01-11

**Authors:** Joshua J Sturm, Kavita Dedhia, David H Chi

**Affiliations:** 1 Department of Otolaryngology, University of Pittsburgh Medical Center; 2 Medical Scientist Training Program, University of Pittsburgh School of Medicine; 3 Department of Otolaryngology - Head & Neck Surgery, Emory University School of Medicine; 4 Department of Pediatric Otolaryngology, Children’s Hospital of UPMC

**Keywords:** cervical thymic cyst, pediatric neck mass, pediatric otolaryngology

## Abstract

We present the case of a 10-year-old boy with the sudden onset of a large, painless left neck mass. Findings on magnetic resonance imaging (MRI) and fine needle aspiration (FNA) biopsy suggest a cystic lesion, most likely of thymic origin. Cervical thymic cysts are a rare form of cervical mass, which are easily overlooked in the differential diagnosis of children presenting with painless neck masses. A combination of CT and MRI investigations can be helpful in differentiating thymic cysts from other congenital and neoplastic masses, but the definitive diagnosis of thymic cyst requires histopathological documentation of thymic tissue. Surgical excision is considered the management of choice for thymic cysts, and no cases of postoperative recurrence have been reported.

## Introduction

Cervical thymic cysts (CTC) are epithelial-lined cysts that most commonly present during the first decade of life, with a slight preponderance towards males [1]. Since CTCs represent less than 1% of cystic cervical masses, they are often overlooked in the broad differential diagnosis of children presenting with painless neck masses. It is important, however, for physicians to be able to efficiently and accurately diagnose CTCs since they can usually be surgically excised with little risk of postoperative recurrence [2].

CTCs generally present as a slow-growing, painless neck mass, and can be accompanied by a combination of stridor, hoarseness of voice, and/or dysphagia [3]. CT, MRI, and histopathological examination are all helpful in the diagnosis of CTC.  On CT, CTCs usually appear as homogeneous masses with thin smooth walls and exhibit smooth, regular enhancement with contrast [4]. On MRI, CTCs are also homogenous, with high signals on T2-weighted images. A definitive diagnosis of CTC generally requires a histopathological examination of the cystic tissue demonstrating the presence of lobulated lymphoid tissue containing Hassall’s corpuscles [4-5]. Here, we present a putative case of CTC found in the superior mediastinum of a young boy.

## Case presentation

A 10-year-old boy presented with sudden onset of a large, painless, left neck mass. There was no history of fever, night sweats, malaise, dysphagia, or difficulty breathing. Aside from an upper respiratory tract infection three weeks ago, he had no recent illnesses and no prior episodes of neck swelling or infection. He had a history of pulmonary hypertension but was otherwise healthy. The patient was up to date on all vaccinations. There was no family history of congenital or acquired cystic lesions. Physical examination of the neck revealed a mass on the left, which was firm but not fluctuant, tender, or erythematous. He had full neck range of motion. Based on these findings alone, we were unable to reach a definitive diagnosis and, thus, decided to proceed with magnetic resonance imaging (MRI) of the neck mass. Informed patient consent was obtained from the child's parent.

**Figure 1:**
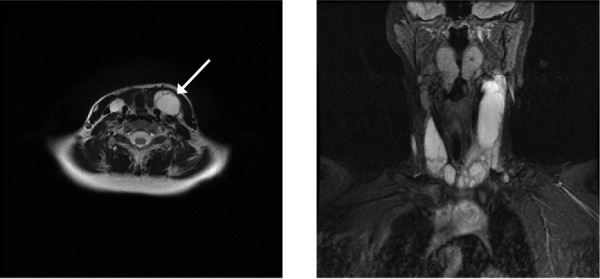
Contrast-enhanced T2-weighted MRI of patient’s neck. Axial (left) and coronal (right) MR imaging of the patient’s neck reveals a peripherally enhancing, multi-septated, cystic lesion arising in the superior mediastinum and extending bilaterally in the neck. The lesion lies deep to the sternocleidomastoid muscles and anterior to the carotid sheaths.

Contrast-enhanced magnetic resonance imaging (MRI) of the neck revealed a peripherally enhancing, multi-septated, cystic lesion that arose in the superior mediastinum and extended into both sides of the neck, deep to the sternocleidomastoid muscles (SCM) bilaterally, and anterior to the carotid sheaths (Figure 1). The left-sided lesion was approximately 12.5 cm in total length, and 3.8 x 2.5 cm in the anteroposterior (AP) and transverse dimensions, while the right-sided lesion measured approximately 9 cm in total length, and 1.9 x 1.6 cm in the AP and transverse dimensions. No additional enhancing lesions were noted.

Ultrasound-guided fine needle aspirations (US-FNA) of the cystic and solid lesions in the left neck each revealed a hypocellular, proteinaceous fluid with few histiocytes, degenerating cells, and inflammatory cells, and without evidence of malignant cells. No definitive evidence of thymic tissue containing Hassall’s corpuscles was obtained. After aspiration of cystic fluid was performed, no apparent neck mass was visible. Further therapeutic options were discussed with the patient’s family (including sclerotherapy, excision, and observation), and they opted to observe and follow up two months later. At his two month follow-up, no increase in swelling was noted, and the patient reported no pain or decreased range of motion. We plan to follow-up again in six months, or sooner if the mass returns.

## Discussion

Cervical thymic cysts are rare, painless neck masses that can present suddenly in children [9]. CTCs can be either congenital or acquired and are most commonly found in the left neck, anterior to the SCM, with approximately 50% showing extension into the superior mediastinum [10]. Unilocular CTCs are most common and are thought to arise due to the persistence of the embryonic thymopharyngeal duct(s). The thymopharyngeal ducts are derived from the ventral surface of the third pharyngeal pouch during the sixth week of intrauterine life, and failure of these ducts to regress during the eighth week of development can cause CTCs to form. Less commonly, patients present with multilocular CTCs, which, unlike unilocular cysts, are thought to result from cystic degeneration of Hassall’s corpuscles contained within the ectopic thymic tissue.

### Differential diagnosis

The differential diagnosis of cervical thymic cyst is broad and includes thyroglossal duct cyst lymphovascular malformations, branchial cleft cyst, and laryngocele, as well as benign tumors (dermoid cysts, epidermoid cysts) and malignant tumors (lymphoproliferative, soft tissue sarcoma and other metastatic lesions) [6]. CT can generally help to differentiate thymic cysts from other cervical lesions, and MRI can be useful in determining the association of putative lesions with the thymus. CTCs are usually found close to the carotid sheath, between the internal jugular vein and the carotid vessels, while branchial cleft cysts are more commonly found superficial and lateral to both the internal jugular vein and carotid artery, and lymphangiomas are generally restricted to the posterior triangle of the neck [7]. While such imaging findings can be useful, the histopathological identification of thymic tissue containing Hassall’s corpuscles is generally necessary to definitely distinguish CTCs from other cervical masses [8].

### Treatment

Surgical excision is the management of choice for CTCs, and there have been no reported recurrences after complete excision in the pediatric population [2]. Most CTCs can be completely excised with a transverse cervical incision [8]. However, it is critical that the existence of a mediastinal thymus be confirmed with MRI or FNAC prior to surgery because thymectomy during childhood can impair immune status later in life [7]. Close postoperative monitoring for evidence of neurovascular compromise (particularly aspiration) is warranted in all patients treated surgically for CTC (2). 

## Conclusions

Cervical thymic cysts are a rare form of cystic mass that can easily be overlooked in the differential diagnosis of painless neck masses in children. CT, MRI, and FNA are all helpful investigations in the diagnosis of cervical thymic cysts, but a definitive diagnosis requires identification of thymic tissue containing Hassall’s corpuscles. If accurately diagnosed, cervical thymic cysts can be effectively managed with surgical excision with little chance of postoperative recurrence.
